# Supplementary material for: Therapeutic Vulnerabilities in FLT3-Mutant AML Unmasked by Palbociclib
Source: Int J Mol Sci. 2018 Dec 11;19(12):3987. doi: 10.3390/ijms19123987 (PMC6321303; doi:10.3390/ijms19123987)
Supplement: Supplementary file 1 [file ijms-19-03987-s001.pdf]

Supplementary Materials

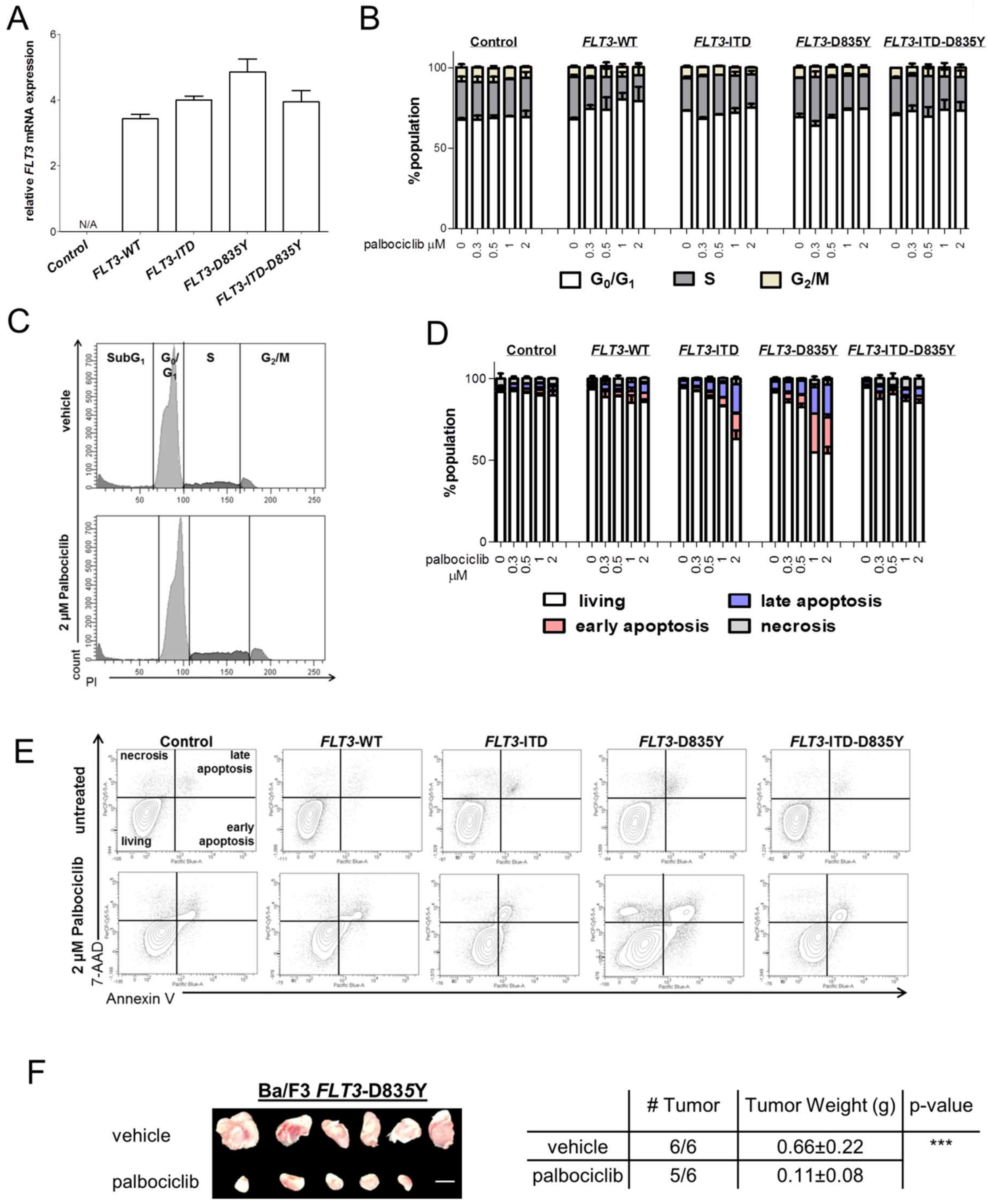

	# Tumor	Tumor Weight (g)	p-value
vehicle	6/6	0.66±0.22	***
palbociclib	5/6	0.11±0.08	

Figure S1. Palbociclib induces apoptosis and impairs *FLT3*-D835Y-driven tumor formation. (**A**) *FLT3* expression was analyzed by quantitative RT-PCR in Ba/F3 cells. Relative expression levels were normalized to *RPLP0* mRNA. Control cells were transfected with empty vector. Error bars indicate ± S.E.M. N/A: not determined. (**B**) FLT3 variants were incubated in the absence of cytokines with palbociclib for 72 hours, stained with propidium iodide and analyzed by flow cytometry. Error bars indicate ± S.E.M. (**C**) Representative PI cell cycle profiles of data shown in Figure 1B and SFigure 1B for control Ba/F3 cells. (**D**) FLT3 variants were incubated in the absence of cytokines with palbociclib for 72 hours, stained with annexin V/7-AAD via FACS. (**E**) Representative flow cytometry contour plots of Annexin-V/7-AAD stained cells as shown in SFigure 1D. (**F**) *FLT3*-D835Y^{+} cells were injected subcutaneously into both flanks of immune-compromised Rag2$^{-/-}\gamma c^{-/-}$ recipients. Mice were treated 3x a

week with vehicle or palbociclib on day 8 (vehicle, n=3 mice; palbociclib, n=3 mice; *** $p < 0.001$) until terminal workup at day 17. Horizontal lines indicates no visual tumor.

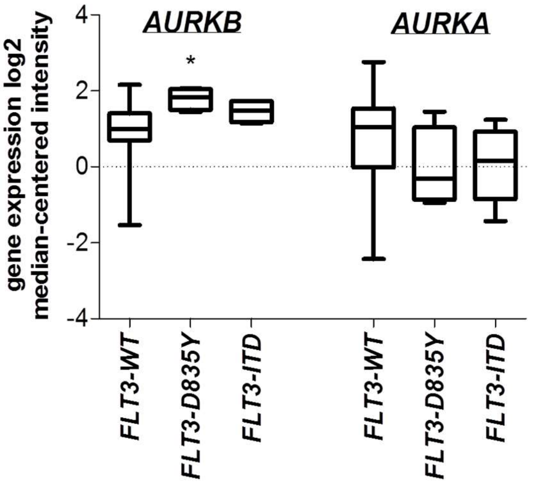

Figure S2. Oncomine analysis of *AURORA kinase* expression in *FLT3*-mutant human cancer cells. * p < 0.0.5.

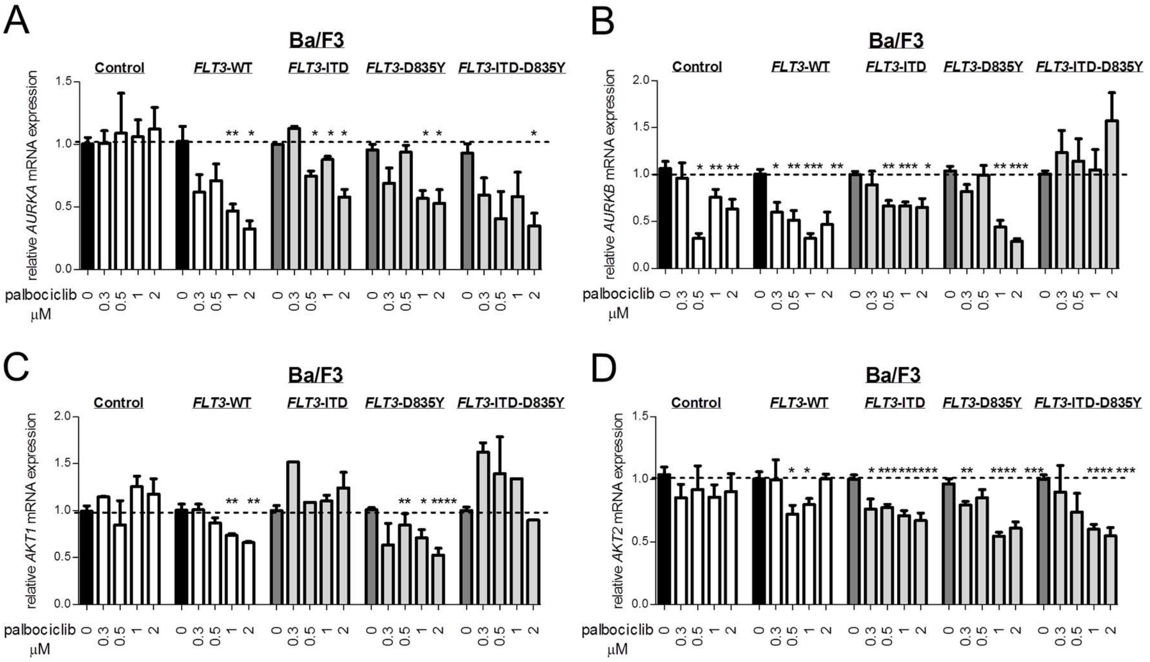

Figure S3. CDK6 regulates expression of *AKT* and *AURORA kinases* in *FLT3*-ITD[+] and *FLT3*-D835Y[+] Ba/F3 cells in a kinase-dependent manner. (**A–D**) Gene expression was analyzed by quantitative RT-PCR in Ba/F3 cells of indicated genoytpe after palbociclib treatment for 72 hours. Relative expression levels were normalized to the housekeeping genes *RPLP0* and *HPRT*. Error bars indicate ± S.E.M. (* p<0.05; ** p<0.01; *** p<0.001; **** p<0.0001).

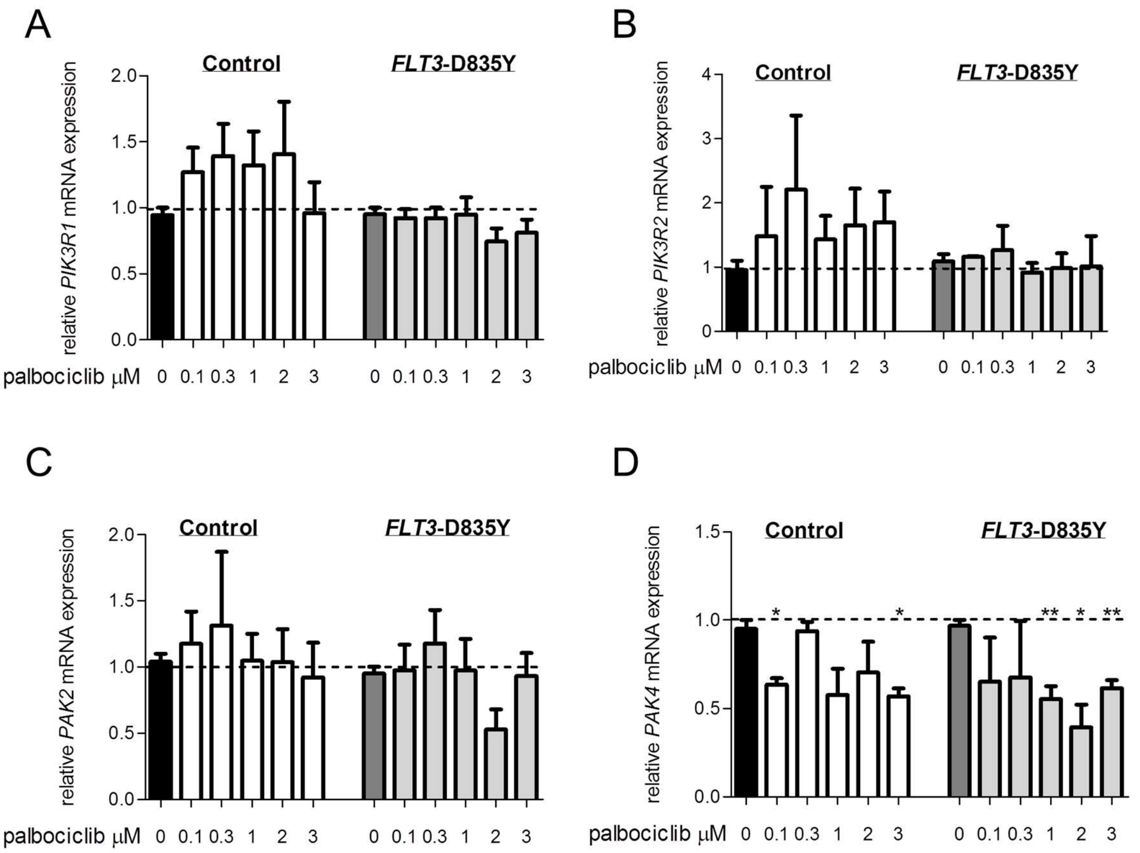

Figure S4. Gene expression analysis upon palbociclib administration in *FLT3*-D835Y⁺ Ba/F3 cells. (**A–D**) Gene expression was analyzed by quantitative RT-PCR in Ba/F3 cells after palbociclib treatment for 72 hours. Relative expression levels were normalized to the housekeeping genes *RPLP0* and *HPRT*. Error bars indicate ± S.E.M. (* $p<0.05$; ** $p<0.01$).

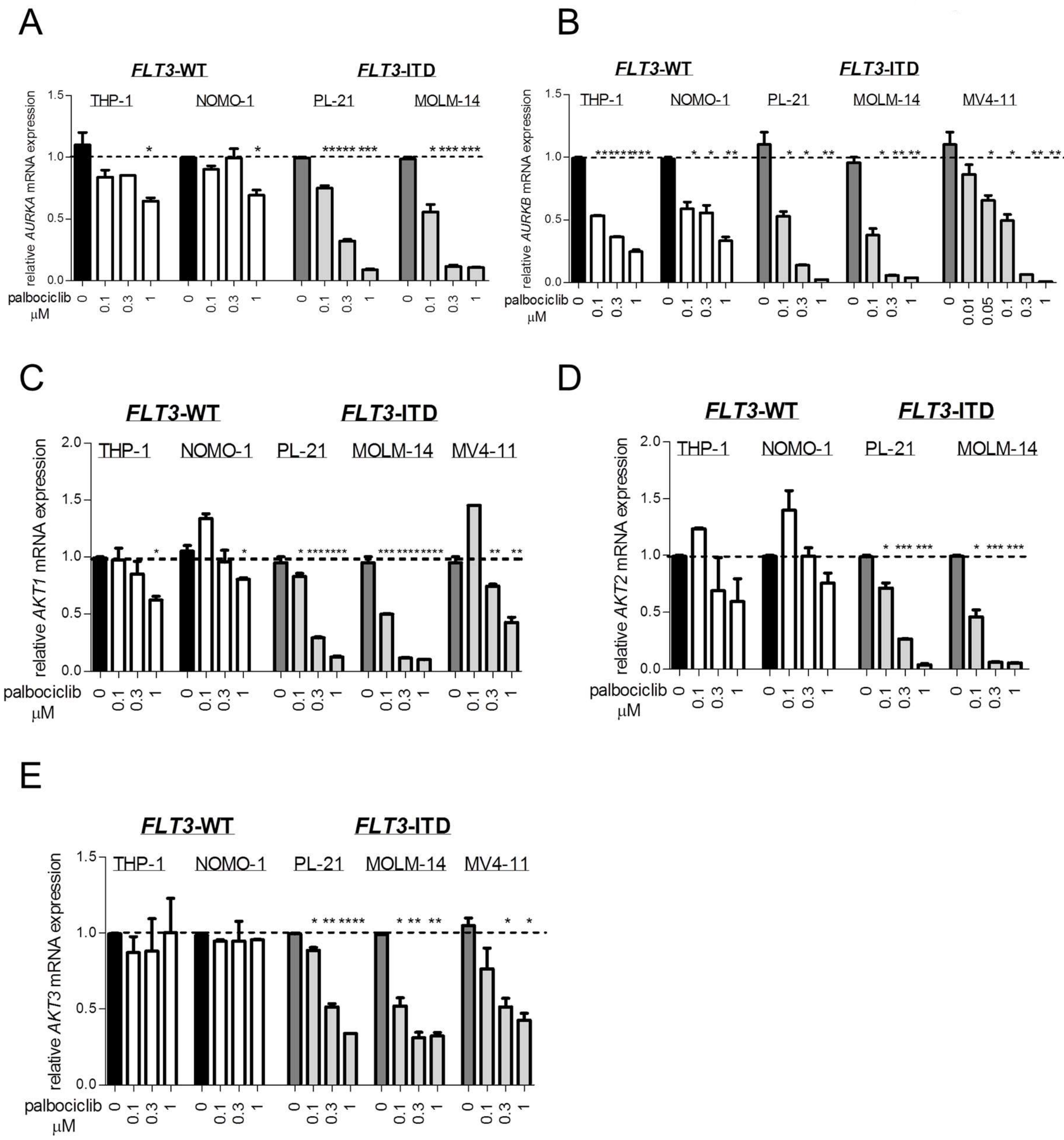

Figure S5. CDK6 regulates expression of *AKT* and *AURORA kinases* in *FLT3*-ITD$^+$ human AML cells in a kinase-dependent manner. (**A–E**) Gene expression was analyzed by quantitative RT-PCR in human AML cell lines of indicated genoytpe after palbociclib treatment for 72 hours. Relative expression levels were normalized to the housekeeping genes *RPLP0* and *HPRT*. Error bars indicate ± S.E.M. (* p<0.05; ** p<0.01; *** p<0.001; **** p<0.0001).

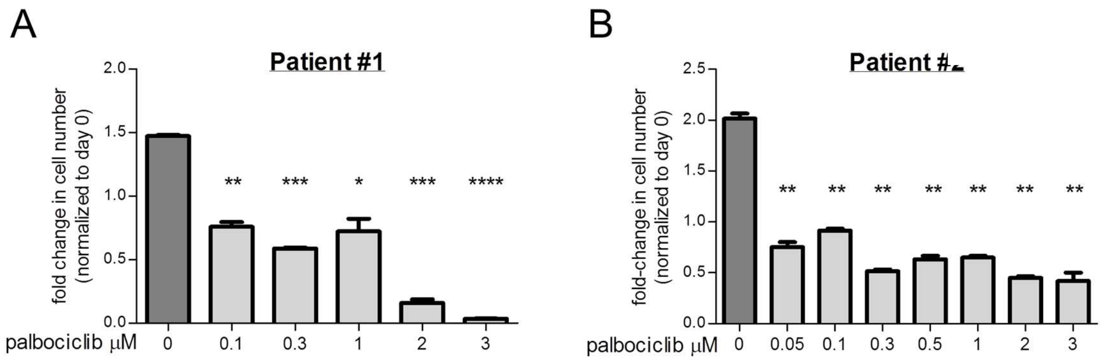

Figure S6. Palbociclib treatment reduces viability of primary *FLT3-D835Y*[+] AML specimens. (**A**,**B**) Primary *FLT3*-D835Y[+] mononuclear cells were subjected to palbociclib. Cell viability was determined by FACS analysis after one week (* p< 0.05; ** p<0.01; *** p<0.001; **** p<0.0001).

A

AURORA kinase inhibitors

**Genentech Cpd10**	***FLT3*-mut**	***FLT3*-WT**
# cell lines	11	878
median IC_{50} (µM)	3.68	11.14
**GSK1070916**	***FLT3*-mut**	***FLT3*-WT**
# cell lines	11	851
median IC_{50} (µM)	1.79	9.52

B

AKT kinase inhibitors

**AKT inhibitor VIII**	***FLT3*-mut**	***FLT3*-WT**
# cell lines	11	878
median IC_{50} (µM)	5	14.736
**AKT inhibitor VIII**	***FLT3*-mut**	***FLT3*-WT**
# cell lines	11	837
median IC_{50} (µM)	5.75	10.12
**GSK690693**	***FLT3*-mut**	***FLT3*-WT**
# cell lines	11	876
median IC_{50} (µM)	20.7	57.4

Figure S7. *FLT3*-mutant human leukemic cells show lower IC_{50} concentrations of AURORA and AKT kinase inhibitors. (**A**,**B**) COSMIC analysis of IC_{50} concentrations of various AURORA and AKT kinase inhibitors in human cancer cell lines. Depicted compounds show selectivity to cells with altered *FLT3* kinase over wild-type *FLT3*-carrying cell lines. Mut: Mutant; WT: wild-type

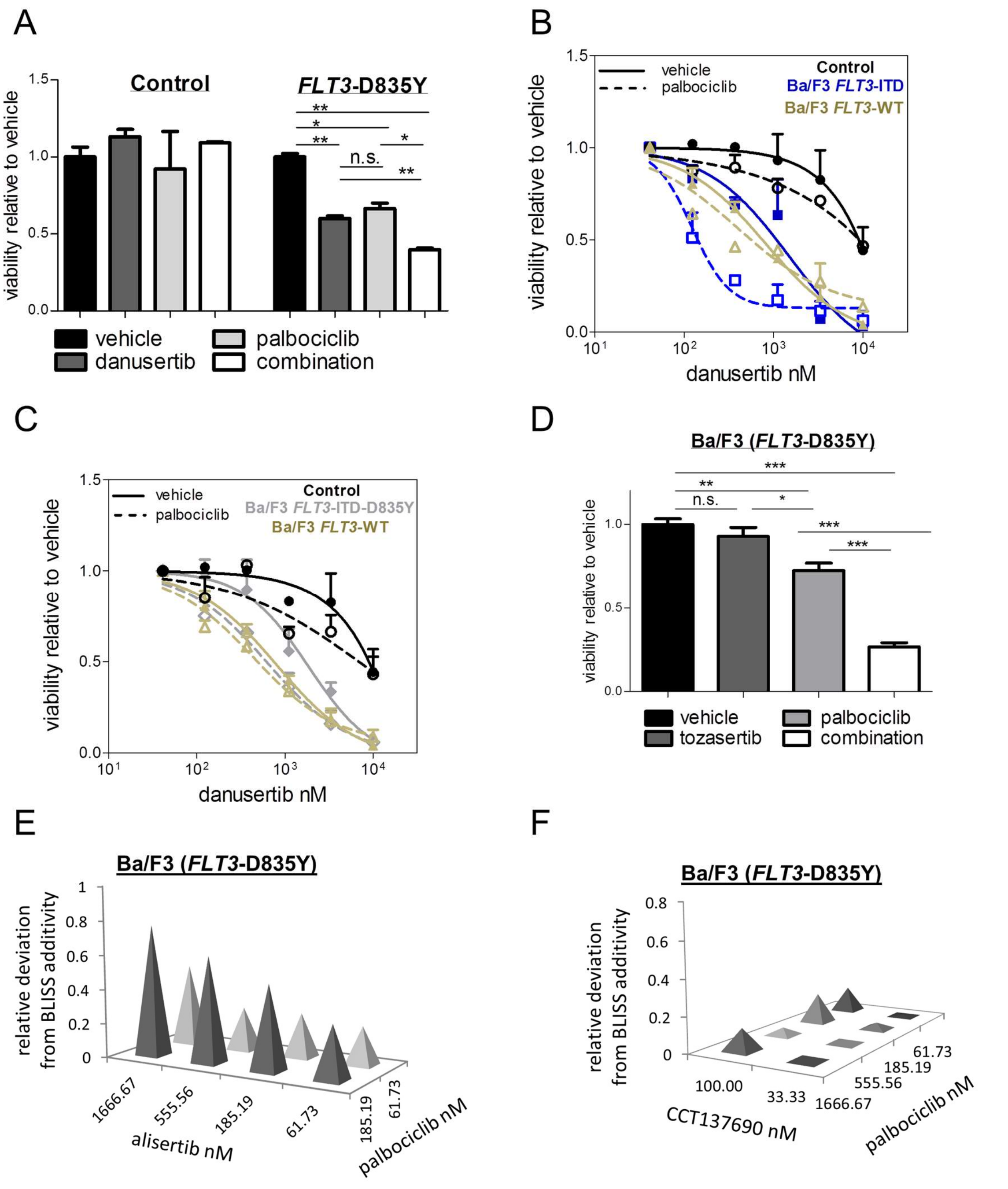

Figure S8. Combined CDK6 and AURK kinase inhibition reveals synergistic effects in *FLT3*-ITD⁺ and *FLT3*-D835Y⁺ Ba/F3 cells. (**A**) Cells were treated with palbociclib (123nM) and the AURORA kinase inhibitor danusertib (123nM) simultaneously or as single therapy. Cell viability and proliferation was assessed by using the CTG assay. Error bars indicate ± S.E.M (n.s.: not significant; * $p<0.05$; ** $p<0.01$). (**B,C**) Dose-response curve with danusertib alone or in the presence of palbociclib (3µM **(B)** and 1µM **(C)** based on the Bliss predicted additivity) in Ba/F3 cells transfected with FLT3 variants. (**D**) Cells were treated with palbociclib (1.6µM) and the AURORA kinase inhibitor tozasertib (61nM) simultaneously or as mono-therapy. Cell viability and proliferation was assessed by using the CTG assay. Error bars indicate ± S.E.M (n.s.: not significant; * $p<0.05$; ** $p<0.01$; *** $p<0.001$). (**E,F**) Combined effects of palbociclib with different AURORA kinase inhibitors. Needle graphs indicate deviation from Bliss-predicted additivity in *FLT3*-D835Y⁺ Ba/F3 cells.

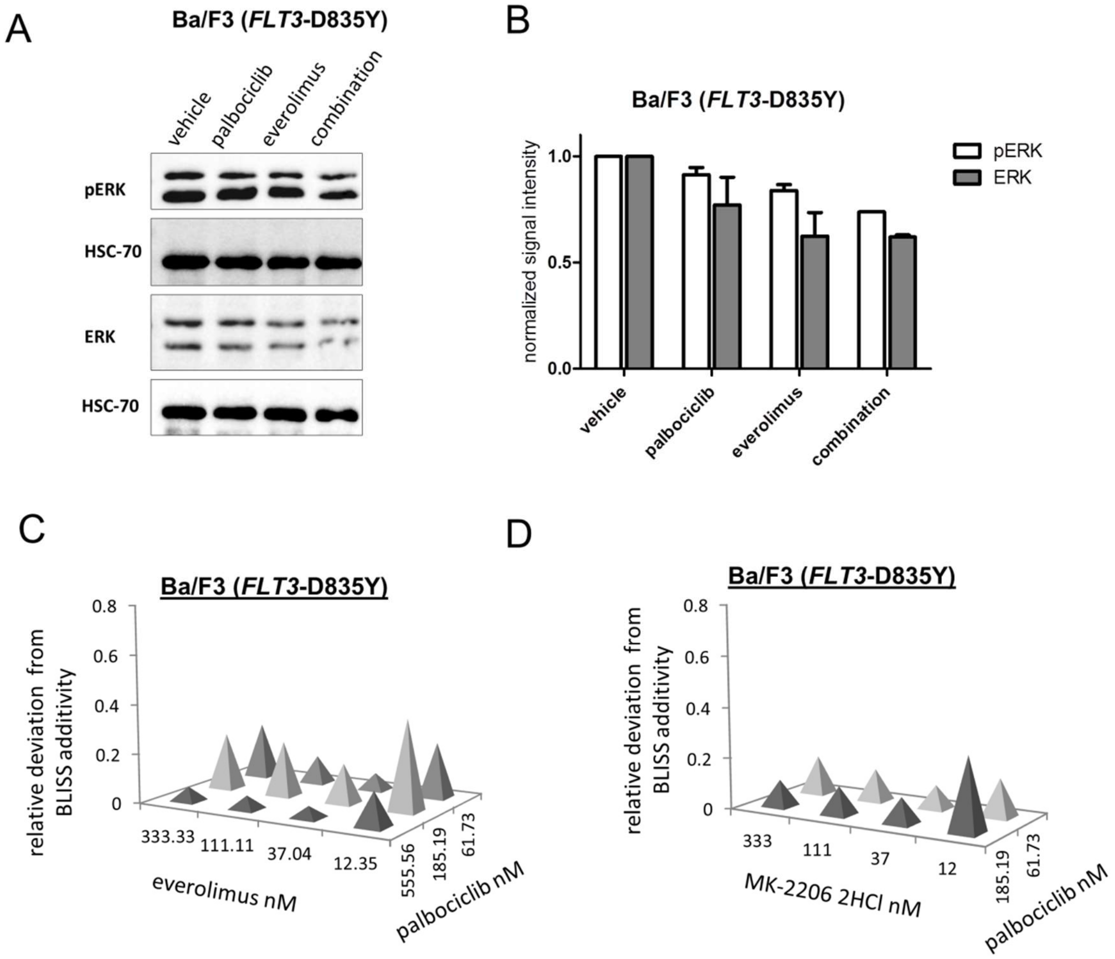

Figure S9. Combined CDK6 and AKT kinase inhibition reveals synergistic effects in *FLT3*-D835Y⁺ Ba/F3 cells. (**A**) Representative immunoblot of Ba/F3 (*FLT3*-D835Y) cells treated for 24 hours with 0.1 μM everolimus, 0.5 μM palbociclib, in combination, or untreated (DMSO control) showing pERK and ERK expression. HSC70 was used as loading control. (**B**) Densitometric quantification of signals in immunoblot shown in (A) normalized to HSC70, n=2. (**C**,**D**) Combined effects of palbociclib with different inhibitors targeting AKT pathway. Needle graphs indicate deviation from Bliss-predicted additivity in *FLT3*-D835Y⁺ Ba/F3 cells.

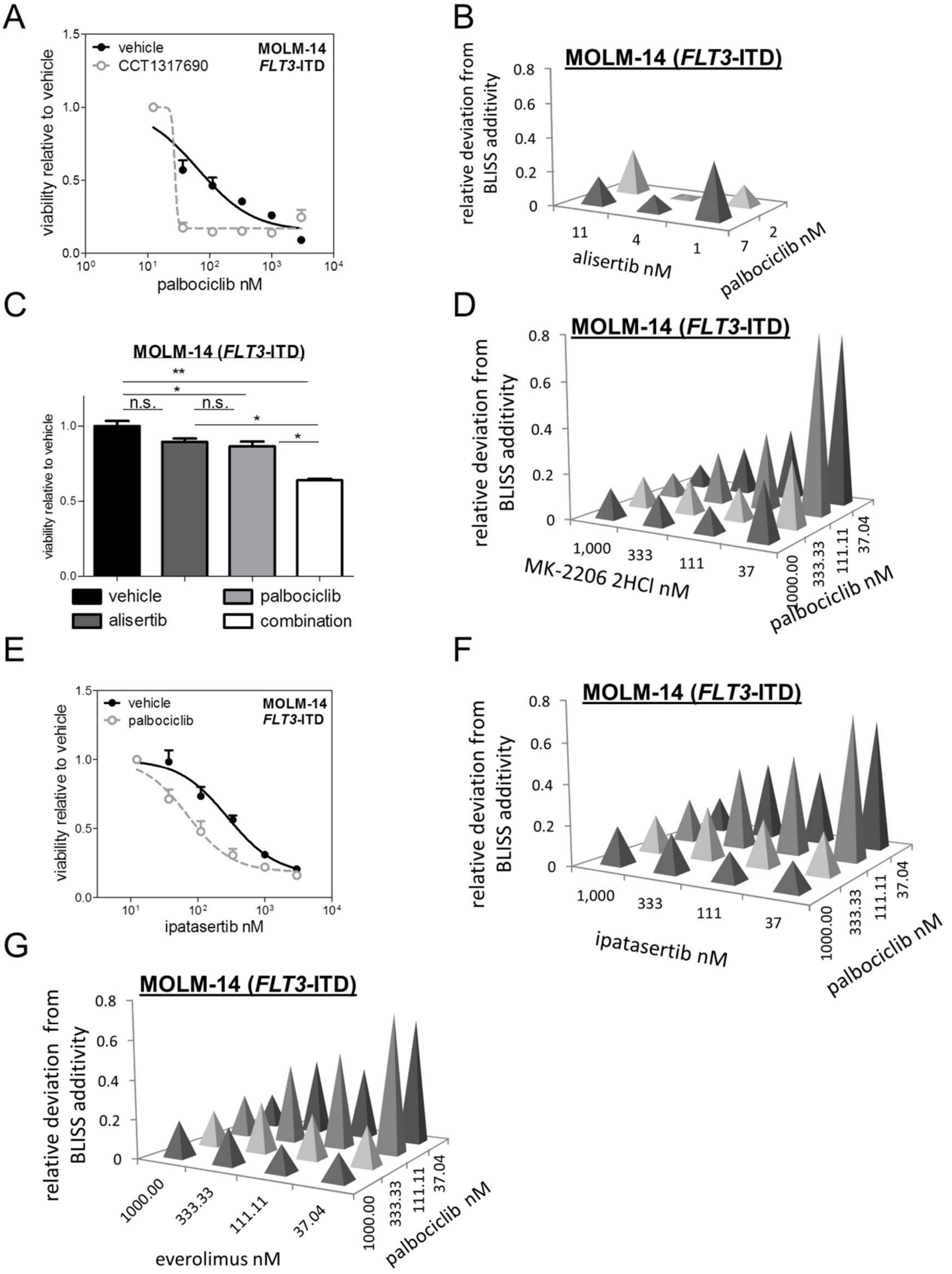

Figure S10. Combined CDK6 and AURK or AKT kinase inhibition reveals synergistic effects in *FLT3*-ITD+ human AML cells. (**A**) Dose-response curve with palbociclib alone or in the presence of the AURORA kinase inhibitor CCT1317690 (37nM based on the Bliss predicted additivity) in the *FLT3*-ITD+ MOLM-14 cell line. (**B**) Combined effects of palbociclib with the AURORA kinase inhibitor alisertib. Needle graphs indicate deviation from Bliss-predicted additivity. (**C**) Cells were treated with palbociclib (2nM) and alisertib (11nM) simultaneously or as single therapy. Cell viability and proliferation was assessed by using the CTG assay. Error bars indicate ± S.E.M (n.s.: not significant; * p<0.05; ** p<0.01). (**D**) Combined effects of palbociclib with the AKT kinase inhibitor MK-2206 2HCl. Needle graphs indicate deviation from Bliss-predicted additivity. (**E**) Dose-response curve with the AKT inhibitor ipatasertib alone or in the presence of palbociclib (0.3μM based on the Bliss predicted

additivity) in the *FLT3*-ITD$^+$ MOLM-14 cell line. (**F-G**) Combined effects of palbociclib with different inhibitors targeting AKT signaling. Needle graphs indicate deviation from Bliss-predicted additivity.
